# Comparative Study of Akin Osteotomies with and Without Fixation in Open Hallux Valgus Correction Surgery: Retrospective Analysis of 200 Osteotomies

**DOI:** 10.3390/jcm15187165

**Published:** 2026-09-15

**Authors:** Enrique Adrian Testa, Annamaria Porreca, Alberto Ruiz-Nasarre, Fernando Alvarez-Goenaga, Daniel Poggio-Cano, Miki Dalmau-Pastor, Carolina Montoya de la Torre, Martin Riegger, Pablo Ruiz-Riquelme

**Affiliations:** 1 Service of Orthopaedics and Traumatology, Department of Surgery, EOC, 6500 Bellinzona, Switzerland; enriqueadriantesta@gmail.com; 2 Master Fellow Foot and Ankle Surgery, University of Barcelona, 08036 Barcelona, Spain; 3 Faculty of Biomedical Sciences, Università della Svizzera Italiana (USI), 6900 Lugano, Switzerland; 4 Human Anatomy and Embryology Unit, Department of Pathology and Experimental Therapeutics, School of Medicine and Health Sciences, University of Barcelona, 08907 Barcelona, Spain; 5 Department of Human Sciences and Promotion of the Quality of Life, University San Raffaele, 00166 Rome, Italy; 6 Unit of Clinical and Molecular Epidemiology IRCCS San Raffaele Roma, 00163 Rome, Italy; 7 Foot and Ankle Unit, Department of Orthopedic and Traumatology, Hospital Sant Rafael, 08035 Barcelona, Spainalvarez173@gmail.com (F.A.-G.); 8 Foot and Ankle Unit, Department of Orthopedic and Traumatology, Hospital Clinic, University of Barcelona, 08036 Barcelona, Spain; 9 Service of Orthopaedics and Traumatology, Department of Surgery, EOC, 6900 Lugano, Switzerland; 10 Foot and Ankle Surgery Unit, Clinica Trinitas and Integramedica, Part of Bupa Chile, Santiago 7500520, Chile; 11 School of Medicine, Finis Terrae University, Santiago 7501015, Chile; 12 Foot and Ankle Surgery Unit, Hospital Clinico La Florida, Santiago 8242238, Chile

**Keywords:** Akin osteotomy, proximal phalangeal osteotomy, closing-wedge osteotomy, hallux valgus

## Abstract

**Background**: In hallux valgus correction surgery, different fixation methods exist for Akin’s osteotomy, but no fixation is also a possibility. We hypothesized that Akin’s osteotomy, both fixed and non-fixed, would provide equally sufficient correction of the phalanx without differences in the measurements of the specific postoperative DASA and IPOA angles and no significant differences between the two groups in the rate of complications. **Methods**: A retrospective single-center comparative case–control study was conducted. Five radiological measurements were analyzed until completion of fusion. The incidence of complications occurring during and after surgery was assessed and compared across the study groups. **Results**: A total of 200 Akin osteotomies were evaluated; 100 were included in the group without fixation and 100 in the group with fixation. Radiologically, the results show that there is no difference in the corrective difference values in the main parameters between the groups. Regarding the analysis of complications, in the group without fixation, all cases achieved fusion. In the group with fixation, one case showed non-symptomatic non-union. Out of 200 operations, four (2%) patients required reoperation. A re-Akin osteotomy was needed for a hypocorrection in the group without fixation. One case in the fixed group went into asymptomatic non-union that did not require re-intervention. **Conclusions**: The absence of internal fixation does not seem to compromise the healing of the osteotomy, and the maintenance of correction grades and complication rates in the group without fixation seems to be similar to the group with fixation. The results are promising; however, we believe that further studies are needed to confirm the data.

## 1. Introduction

Akin’s closing-wedge osteotomy for the treatment of hallux valgus (HV) was first described in 1925 and despite the many options described over the years to maintain the correction achieved, none has proved superior to the other in terms of clinical and radiological results [1].

Over recent years, especially with the rise in minimally invasive and percutaneous procedures, techniques that do not involve fixation have also emerged [1,2,3,4,5,6,7,8,9,10,11,12,13,14,15].

In this context, techniques were performed primarily according to the surgeon’s preference and cultural background and not according to evidence-based choices. We can therefore find examples of fixation with screws, staples, transosseous sutures and, in minimally invasive osteotomies, no fixation at all [1,5,7,10,11,14,15].

However, few studies describe the results related to the correction performed with Akin’s osteotomy, the maintenance of this correction and the complications related to this procedure, and even fewer studies compare different techniques [1,5,10].

An important gap in the literature is the lack of biomechanical studies comparing screw fixation, nitinol staple fixation, transosseous suture fixation, and no fixation within the same Akin osteotomy model, while maintaining identical osteotomy geometry and lateral cortical integrity, and assessing stiffness, displacement, cyclic loading, and ultimate load to failure. Existing studies, including the most relevant biomechanical investigation by Chacon et al., compare only some of these fixation methods, are often conducted using synthetic bone (sawbone) models, and appear to show no clear biomechanical superiority of screws, staples, or sutures over one another [10].

This study aims to compare two groups of patients undergoing Akin osteotomy, one with fixation and a second without fixation, to see if there are differences in postoperative angular changes. Secondly, we will compare the rates of complications related to the surgery. Our starting assumption is that there will be no statistically significant differences between the two groups, with non-inferior angular correction in the phalanges of the non-fixation group and similar complication rates, including non-union and secondary displacement.

## 2. Materials and Methods

A retrospective single-center case–control study was conducted. All cases were taken from a database of foot and ankle units, divided between those with staple fixation (SFA) and fixation-free (FFA) osteotomies, and then randomly selecting 100 from each group. We used the program “Randomizer” (https://www.randomizer.org/, version 4.0). If the selected case also had a correction procedure on the opposite side, this was also included if it met the inclusion criteria. If a case did not meet the inclusion criteria, a new case was drawn. All cases were performed by the clinic’s two senior surgeons (FAG, ARN) or their attending surgeons under their supervision.

Eligible patients were adults aged > 18 years with no history of previous hallux surgery who underwent Akin osteotomy between 2011 and 2018 as part of an open HV correction procedure, irrespective of deformity severity or the type of associated metatarsal osteotomy. Inclusion also required preoperative clinical and radiographic assessment and at least two postoperative radiographic evaluations, performed at 2–6 weeks and 8–12 weeks, respectively. In all cases, radiographic follow-up was continued until complete bone union was documented, defined as osseous bridging of at least three of four cortices across the osteotomy site on the two radiographic projections, according to the criteria described by Rush et al. and McClelland et al. [16,17]. In cases of delayed union, serial radiographic assessments were required until complete radiographic healing was documented. Exclusion criteria comprised a history of previous hallux valgus (HV) or first-ray surgery, age < 18 years, percutaneous procedures, metatarsophalangeal (MTP) arthrodesis, phalangeal osteotomies other than the Akin procedure (e.g., Moberg osteotomy), and previous fractures involving the first ray.

**Figure 1:**
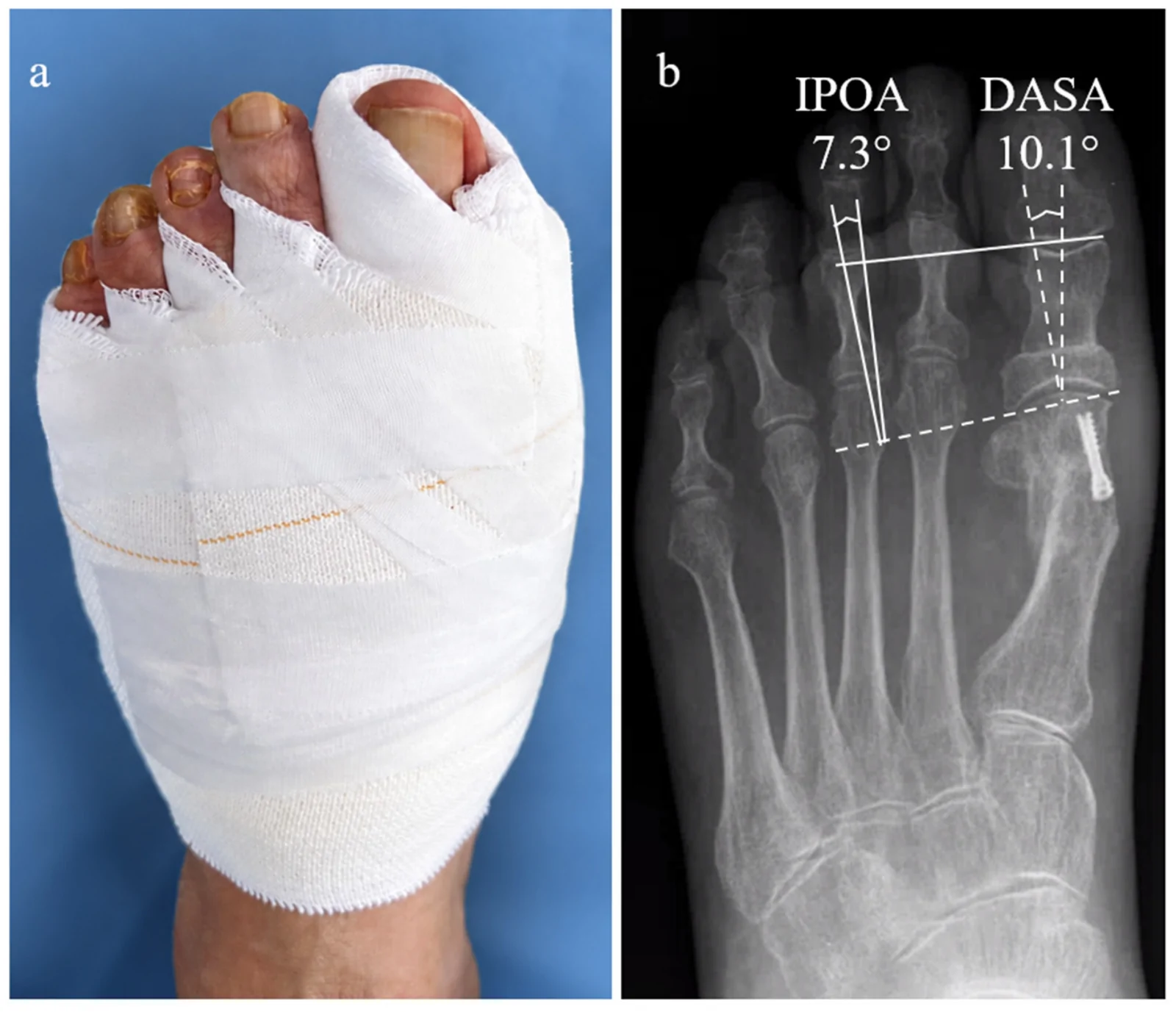
Postoperative bandage and X-ray. (**a**) Corrective postoperative bandage with varus thrust; the figure shows three gauze strips wrapped around the great toe and a single gauze strip around each of the lesser toes. The gauze strips are wrapped inward, creating a spacer effect between the toes while simultaneously applying a varus-directed corrective force. They are maintained in position using a semi-rigid double-sided adhesive bandage, and additional varus-directed adhesive strips are applied to complete the dressing. (**b**) Distal articular set angle (DASA—dashed lines) and interphalangeal joint obliquity angle (IPOA—solid lines) both measured on postoperative X-ray.

The two angular parameters primarily affected by the Akin osteotomy (Figure 1)—the distal articular set angle (DASA) and the interphalangeal joint oblique angle (IPOA)—were assessed preoperatively and at 2–6 and 8–12 weeks postoperatively, with additional measurements obtained until union when required. The hallux valgus angle (HVA), first–second intermetatarsal angle (IMA), and interphalangeal angle (IPA) were also recorded. All angular measurements were performed using the RAIM-Viewer v2.8 digital system. Measurements were initially obtained to one decimal place and subsequently rounded to the nearest whole degree, with decimal values ≥ 0.5° rounded up and those < 0.5° rounded down. Radiographic complications, including delayed union, non-union, and malunion, were assessed and documented. In addition, all intraoperative and postoperative complications were recorded.

Patients without radiographic evidence of osteotomy union at 8–12 weeks underwent continued clinical and radiographic monitoring until consolidation was confirmed, and the time to union was documented. Delayed union was defined as failure to achieve fusion within 12 weeks, whereas non-union was defined as absence of fusion beyond 36 weeks [17,18,19].

Disruption of the lateral cortex occurring either intraoperatively during osteotomy or identified on postoperative radiographs was documented. Additional radiographic outcomes, including delayed union, non-union, malunion, osteonecrosis, algodystrophy, excessive callus formation, and any other osseous abnormalities, were also assessed and recorded. All reoperations were assessed, and those specifically related to complications involving the proximal phalanx were documented.

### 2.1. Surgical Technique

**Figure 2:**
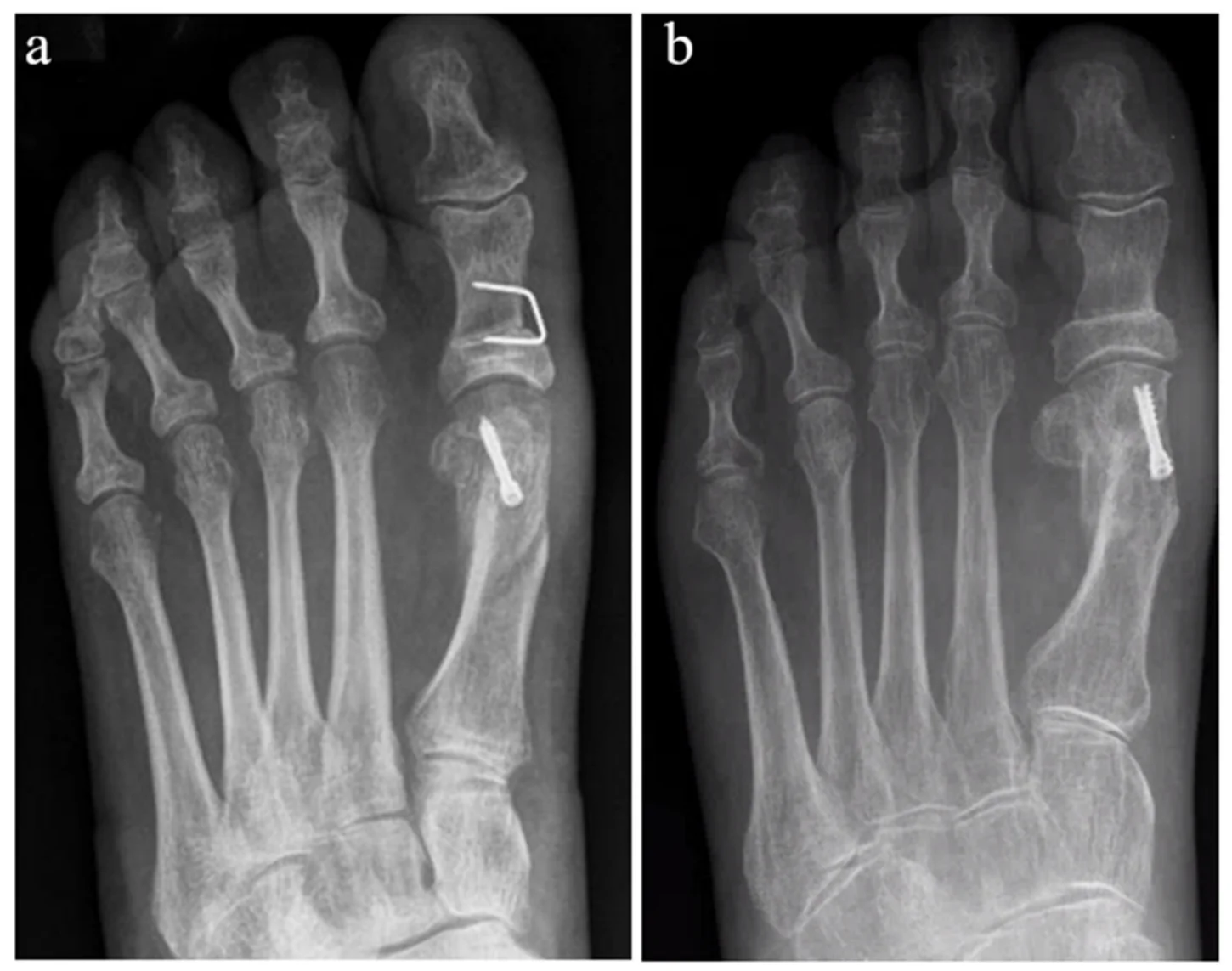
Example of hallux valgus correction with staple fixation (**a**) and without fixation (**b**).

For both techniques, after completing the medial approach and the procedure on the first metatarsal, the phalangeal osteotomy was performed with careful soft-tissue dissection, paying particular attention to protection of the extensor hallucis longus (EHL) and flexor hallucis longus (FHL) tendons. At the proximal metadiaphyseal level, typically 8–10 mm distal to the joint line, an initial cut was made medially to laterally, parallel to the joint surface. A second cut was then initiated approximately 2–4 mm proximal to the first and directed laterally and proximally until the two osteotomy lines converged. During both cuts, care was taken to preserve the integrity of the lateral cortex. The width of the osteotomy wedge was determined according to the intended degree of correction, taking into account the calculations described by Frey et al. and the clinical appearance of the hallux following correction [20]. Once the bone wedge is removed, the gap is closed manually. Then, if the operator has planned to fix the osteotomy, it is performed by means of a staple positioned so that the osteotomy cut is equidistant to the two legs of the staple, while maintaining the wedge closure manually as well as a tensioned medial capsule closure (Figure 2). If, on the other hand, fixation was not planned, then a tensioned medial capsule closure was performed. The dressing was applied using three elongated gauzes wrapped around the great toe in a tie-like configuration, providing additional varus-directed traction. An elongated gauze strip was subsequently applied to each toe to maintain a varus-directed position. Adhesive strips were placed over the gauze to preserve gentle tension while avoiding skin injury (Figure 1). Immediate full weight-bearing was permitted using a rigid-soled postoperative shoe for 4–6 weeks.

### 2.2. Statistical Analysis

In this retrospective study, we compared observation surgery outcomes from 2 techniques before (T0) and after (T1) surgery. Descriptive statistics are presented as mean and standard deviation (SD) for continuous variables and absolute frequency (*n*) and percentage (%) for categorical variables. The *t*-test was used to assess the mean difference between independent samples, while the association between group membership (FFA or SFA) and categorical variables was evaluated using the Chi-squared test (for cell frequency *n* ≥ 5), or Fisher’s exact test (for cell frequency *n* < 5). Mixed-effect models using patient id as a random effect were computed to include the hierarchical nature of the analysis. All statistical tests were 2-sided with a significance level set at *p*  ≤  0.05. All analyses were performed with the open-source statistical R environment (Version 4.3, R Foundation for Statistical Computing, Vienna, Austria). Because a significant baseline difference was identified in preoperative DASA, an additional regression-based sensitivity analysis was performed with DASA at 3 months as the dependent variable and fixation strategy and preoperative DASA as explanatory variables. The assumption of homogeneity of regression slopes was assessed by testing the interaction between preoperative DASA and fixation strategy. Because this interaction was statistically significant, the interaction term was retained in the final model rather than applying a conventional ANCOVA assuming parallel regression slopes. Regression coefficients, 95% confidence intervals, *p* values, and partial eta squared (η^2^p) were reported. Furthermore, because the distribution of concomitant first-metatarsal osteotomies differed between groups, an additional sensitivity analysis was performed including metatarsal osteotomy type (Chevron versus Scarf) as a covariate. The single patient who underwent bunionectomy without Chevron or Scarf osteotomy was excluded from this analysis, resulting in a cohort of 199 patients. Separate adjusted models were constructed for DASA, HVA, and IMA at 3 months, including the corresponding preoperative value, fixation strategy, and metatarsal osteotomy type. For DASA, the previously identified interaction between preoperative DASA and fixation strategy was retained in the model.

### 2.3. Ethical Statement

The study protocol, including the anonymization of patient data, received approval from the Institutional Review Board (IRB; Ethics Committee PR-2023-16). As an observational investigation, the study involved no experimental procedures performed by the authors on either human participants or animals.

For the illustrative radiographs (Figure 1 and Figure 2), informed consent was obtained.

## 3. Results

Between 2011 and 2018, we identified 875 interventions in the first ray, of which 473 were primary HV corrections that also included an Akin osteotomy within the foot and ankle unit. Of these, 187 were performed with staple fixation and 286 without fixation. The decision to fix or not fix Akin’s osteotomy depended on the surgeon’s preference and not on the severity of the degree of hallux valgus or other clinical or radiological factors.

For each group, we then extracted 100 cases using the Randomizer program. We arbitrarily chose to have 100 feet per group. A total of 74 patients met the inclusion criteria in the FFA group while 68 met the criteria in the SFA group. All cases of bilateral procedures were performed at different surgical times.

We found a good homogenization of the groups. Table 1 shows the demographic characteristics for the two groups. Regarding the associated first-metatarsal procedures, bunionectomy was performed in one patient in the non-fixed group and none in the fixed group, Chevron osteotomy in 81 and 69 patients, respectively, and Scarf osteotomy in 18 and 31 patients, respectively.

Table 2 shows the mean angular differences in the five parameters considered in the two groups between preoperative and control after fusion. The mean values are accompanied, in brackets, by standard deviation (SD).

At the time of radiographic union, the mean change in DASA from the preoperative value was 6.40° in the FFA group and 7.45° in the SFA group (Table 2). However, according to Table 3, this difference was not statistically significant (*p* > 0.05), and no significant interaction between time and group was found, indicating that the change in DASA over time followed a similar pattern in both groups.

Similarly, we found an average difference between pre-op and post-op at the fusion time of IPOA of 10.40° for the FFA group and 8.35° for the SFA group (Table 2). Table 3 shows that this difference was not statistically significant (*p* > 0.05), and the interaction term was also not significant, suggesting that the evolution of IPOA over time was comparable between the groups.

IPA shows a variation of −6.18° in the FFA group and −7.90° in the SFA group (Table 2). According to Table 3, the interaction effect between time and group was significant (*p* < 0.01), indicating that the rate of change over time was different between the groups. Although its clinical significance remains uncertain, the observed interaction may reflect differences in osteotomy size or variations in postoperative management, particularly bandaging techniques, which could have influenced changes in IPA over time. These potential confounding factors should therefore be considered when interpreting this result.

In contrast, the differences observed in HVA and IMA, just as for DASA and IPOA, were statistically significant over time (*p* < 0.001, Table 3), but the interaction terms were not significant. This indicates that while these variables changed significantly from pre-op to post-op, the pattern of change was similar in both groups (IMA: SFA vs. FFA = 8.01° vs. 7.85°, *p* > 0.05; HVA: SFA vs. FFA = 23.9° vs. 21.8°, *p* > 0.05; Table 3).

A significant interaction was identified between preoperative DASA and fixation strategy (β = −0.402, 95% CI −0.719 to −0.086; F[1, 196] = 6.28; *p* = 0.013; partial η^2^ = 0.031), indicating that the relationship between preoperative and postoperative DASA differed between the groups. The estimated slope relating preoperative DASA to DASA at 3 months was 0.164 in the fixation group and 0.566 in the non-fixation group. Consequently, a conventional ANCOVA assuming homogeneous regression slopes was not applied. At the overall mean preoperative DASA of 2.03°, the estimated adjusted between-group difference in postoperative DASA was 1.30° (95% CI −0.07° to 2.67°; *p* = 0.063).

In the sensitivity analysis including 199 patients undergoing either Chevron or Scarf osteotomy, the type of concomitant metatarsal osteotomy was not significantly associated with DASA, HVA, or IMA at 3 months. For DASA, the interaction between preoperative DASA and fixation strategy remained significant after adjustment for metatarsal osteotomy type (β = −0.421, 95% CI −0.738 to −0.104; *p* = 0.009), whereas Scarf versus Chevron osteotomy was not associated with postoperative DASA (β = −0.48°, *p* = 0.514). At the mean preoperative DASA, the adjusted fixation versus non-fixation difference was 1.26° (95% CI −0.13° to 2.64°; *p* = 0.076).

For HVA, metatarsal osteotomy type was not significantly associated with the postoperative value (β = −0.50°, *p* = 0.595), while fixation strategy was associated with a 2.09° higher HVA at 3 months after adjustment for baseline HVA and osteotomy type (95% CI 0.50° to 3.68°; *p* = 0.010). For IMA, neither metatarsal osteotomy type (β = 0.48°, *p* = 0.197) nor fixation strategy (β = 0.26°, 95% CI −0.37° to 0.89°; *p* = 0.418) was significantly associated with the postoperative value.

Table 4 shows the intra- and postoperative complications in the two groups.

The lateral cortex is shown to be disrupted in 42% of cases in the FFA group and in 15% of cases in the SFA group (*p*-value < 0.01).

The SFA group shows one case of non-union while the FFA group has no cases. It is noteworthy that the patient with non-union was not symptomatic and did not want to undergo further clinical or radiographic examination.

There was delayed union in 10% of the cases in the FFA group with a mean fusion time of these delayed cases of 26.2 weeks compared to 2% in the SFA group (*p*-value < 0.05), with a mean fusion time of these delayed cases of 24 weeks.

Only one case in the SFA group showed the development of a hypertrophic callus that required no treatment. No excessive calluses were observed in the FFA group (*p*-value > 0.05).

In 6% of the cases in the FFA group, we observed a malunion. In all cases, it presented as a slight extension of the distal fragment with plantar flexion of the osteotomy of less than 10°. These deviations were clinically asymptomatic and did not require any treatment. No malunion cases were present in the SFA group (*p*-value < 0.05).

Three cases required surgery in the FFA group, two for recurrence of hallux valgus and one for superficial wound infection. Of the two cases of recurrence, only one required a surgical reoperation of the Akin osteotomy. In the SFA group, only one case required reoperation for superficial soft-tissue infection.

Table 5 shows the association between lateral cortex interruption and delayed union and malunion in SFA and FFA groups: no statistically significant differences were found between the groups.

## 4. Discussion

This is a comparative study of Akin’s osteotomy with and without fixation in the surgical treatment of hallux valgus. Other comparative studies with different techniques have been published, but to our knowledge, to date, this is the largest study comparing a staple fixation technique with a group without fixation. The most important conclusion of our analysis is that the correction given by Akin’s osteotomy is adequately maintained regardless of the group (fixed or unfixed) and that the risk of severe complications is essentially overlapping in the two groups. Negligible complications that do not require any kind of treatment, such as asymptomatic delayed union or malunion of less than 10°, appear to be more common in the FFA group, while the advantages of not having complications related to the use of material and the reduction in costs are undeniable.

Our data seem to confirm those of a recent study by Zaragosa et al. who compared 89 patients divided into two groups, 59 fixed with a cross-suture configuration of the joint capsule and 30 fixed with staples; Akin’s osteotomy with fixation using metal implants produced radiological results comparable to suture fixation methods without an increase in associated accidents [21].

Other articles discuss the possibility of fixing the osteotomy with transosseous sutures [1,5,9,22,23].

Recently, Matsumoto et al. compared a case series of patients with staple fixation with a series of cases fixed with transosseous Fiberwire suture; in this study, the cases treated with osteosuture appeared to demonstrate superiority in terms of stability and cost-effectiveness [22].

Sinnett et al., in a comparative study of transosseous and staple suture, showed that there were no significantly differences in results for all outcomes considered between the two groups, except for cost in favor of the suture group [5].

Liska et al. carried a comparative study of Akin’s osteotomies fixed with staples vs. screws vs. sutures and showed how the results of the suture group were comparable with the other types of fixations; it was also shown how the procedure is cost-effective and avoids complications related to the use of metal material [1].

### 4.1. Angular Correction

Regarding the angular corrections of the two reference angles (DASA and IPOA), our study indicates that the correction values obtained are comparable regardless of the baseline parameters. This suggests that the maintenance of correction is not influenced by the initial degree of deviation (Table 3).

We believe that the use of the maintenance bandage is a fundamental part of the therapy and, as Liska already pointed out in a note to the authors, is the real key to successful treatment, more so than the type of capsular tension suture or transosseous suture or even more likely a combination of the two [24]. To date, we cannot give a clear answer to this question with the present study. However, our data aligned with all the comparative studies analyzed, supporting the idea that the outcomes considered are not influenced by the type of fixation used and that intrinsic fixation using osteosynthesis material is not strictly necessary for the osteotomy to heal [1,3,5,21,22].

### 4.2. Complications

#### 4.2.1. Delayed Union/Non-Union

Non-union rates range between 0.0% and 2.2% and delayed union rates between 3.4% and 28.3% [12,13,14,20,25,26,27,28,29].

An interesting point to highlight is that in our study, in the group without fixation, there were no cases of non-union, which is in line with the few studies published to date dealing with Akin’s osteotomy fixed with tension capsule suture or trans-bone suture [1,5,21,22].

As already observed in a recent case series, we confirmed that in a substantial percentage (10%) of cases in the group without fixation, a radiological consolidation delay is observed [6]. These are in all cases of asymptomatic delays where complete radiological closure was observed at an average of 26.2 weeks.

This data contrasts with some publications describing similar data in percutaneous Akin surgery without fixation, like that in Herrera Perez et al. who showed complete fusion at 8 months in nine of 26 delayed union cases [13,29].

In both groups (SFA and FFA) we observed a statistically significant correlation between lateral cortical breakage and delayed union (Table 4). This data is in line with two recent studies by Testa et al. and Matsumoto et al. [6,22].

#### 4.2.2. Malunion

For what concerns a union in an incorrect position, rates between 0.0% and 24.0% have been described in the literature [14,20,27,30]. In six cases (6%) in the FFA group, we observed healing in mild extension (<10°) that was completely asymptomatic and did not require any type of treatment, while no cases were observed in the group with fixation.

We believe that leaving the lateral cortex intact can ensure greater stability, whilst avoiding the risk of abnormal consolidation [4,11,12]. The present data cannot confirm this theory. Malunion was observed in six cases in the FFA group and 0 cases in the SFA group. Of the six cases, five had lateral cortical penetration while one did not, but the difference between the groups was not statistically significant (*p* = 0.091). The six cases had a deviation of less than 10°, without any discomfort or functional limitations, so none required a second operation for this problem.

#### 4.2.3. Lateral Cortical Disruption

Douthett et al. observed a disruption of the lateral cortex in 47/132 (35.0%) cases [12]. Schilde, comparing cases of Akin performed percutaneously with open surgery, described a lateral cortical disruption in 13.9% of the open group and in 51.6% of the minimally invasive group [3]. Matsumoto reported 10.3% lateral cortical disruption in 97 operated cases with no difference between the staple-fixed and non-fixed groups [22].

In our case series, we found a higher rate of lateral cortical disruption in the group without fixation (42%) than in the group with fixation (15%), aligning with Schilde’s study [3]. We have interpreted this difference with two possible explanations: First, the lack of stable fixation may cause the load to spontaneously complete the osteotomy at the first steps. We believe this to be unlikely, given the direction of the forces at the load and the use of a hard-soled shoe in our protocols. An alternative explanation could be the interpretation of the postoperative radiographs. A complete osteotomy of the lateral cortex but firmly fixed medially could have no dislocation and this would be undetectable on radiographic control at 6 weeks. On the other hand, a complete osteotomy without fixation would cause even a minimal dislocation to be detected on the radiographic controls.

Interestingly, we found a significant association between lateral cortex disruption and delayed union in the fixation-free group (Table 5). Patients with cortex disruption had a delayed union rate of 19% compared to 3.4% in those without disruption (*p* = 0.026). This suggests that preservation of the lateral cortex is critical for successful union in non-fixed Akin osteotomies. Surgeons should be meticulous in protecting the lateral cortex during the osteotomy.

### 4.3. Others

In our cases, we did not experience any staple disturbance, although one case from the SFA group presented a wound disturbance problem with a hypertrophic and painful wound that required a revision of the surgical wound. The scar was excised 3 years after surgery and the staple was not removed with complete regression of symptoms. Garrido recently presented a case history with 6.3% (two cases) of staple disorders requiring removal of the means [31]. Similarly, Liska reported staple disturbances in one case out of 43 [1]. In other cases, history with staples did not present this type of problem [5,32].

As already described in one of our previous publications, we believe that the advantages of no fixation are clear: saving surgical time; absence of material-related complications such as non-tolerance of the material, migration, perforation of the joint, or irritation of the tendons; and finally, surgery is more cost-efficient as the cost of the material is avoided (and the possible additional cost of material removal) [6]. Previous studies evaluating Akin osteotomy fixation have reported implant costs of approximately $75 for a headless compression screw and $80–130 for staple fixation, compared with $3–36 for suture fixation [1].

### 4.4. Limitations

These findings should be interpreted cautiously given the retrospective case–control design and the inherent methodological limitations and potential sources of bias associated with this type of study.

The most important limitation of this study is its retrospective design, which precluded the collection of functional outcome scores, such as the AOFAS score. Therefore, our findings should be interpreted primarily in terms of radiographic correction and should not be directly extrapolated to clinical outcomes. Future prospective studies should address this limitation by evaluating both radiographic outcomes and validated functional and patient-reported outcome measures.

Another limitation of this study is that it does not fully isolate the effect of Akin osteotomy fixation, as patients underwent different concomitant metatarsal procedures that may have independently influenced the outcomes.

The present findings should be interpreted in light of the retrospective and non-randomized study design. Allocation to fixation or non-fixation was based on surgeon preference and may therefore have introduced selection bias and confounding by indication. Although the groups were comparable in terms of the main parameters reflecting overall hallux valgus severity, including HVA and IMA, a significant baseline difference was observed for DASA. An additional adjusted analysis was therefore performed to account for this imbalance. Notably, a significant interaction between preoperative DASA and fixation strategy was identified, indicating that the relationship between baseline and postoperative DASA differed between the two groups, precluding the use of a conventional ANCOVA assuming homogeneous regression slopes. The interaction was therefore retained in the final model. Furthermore, potentially relevant factors such as bone mineral density and standardized measures of patient activity were not systematically recorded and could not be reliably reconstructed retrospectively. Consequently, although the additional analysis accounts for the observed baseline DASA imbalance, residual confounding from unmeasured factors cannot be excluded. These findings should therefore be interpreted as associations within a retrospective cohort and not as evidence of formal equivalence between fixation and non-fixation strategies.

Another potential source of confounding is the different distribution of concomitant first-metatarsal osteotomies between the groups. Since Chevron and Scarf osteotomies independently contribute to HVA and IMA correction and may influence overall forefoot mechanics, metatarsal osteotomy type was included as a covariate in an additional sensitivity analysis. Although osteotomy type was not significantly associated with postoperative DASA, HVA, or IMA in the adjusted models, concomitant procedures were not randomized and their contribution to the overall radiographic and biomechanical outcome cannot be completely separated from that of the Akin osteotomy. Residual confounding related to procedural selection therefore remains possible.

The limitations of measurements, as described by Camak et al., are because in most cases the rotation of the phalanges can influence some angular parameters in the standard two-dimensional projections in the context of HV [33]. Currently, the limits of three-dimensional measurements, which are certainly more reliable, are due to possible additional irradiation or excessive costs.

Finally, it should be emphasized that all the Akin osteotomies described in the present study were performed using an open surgical approach. Therefore, although our findings are consistent with the trends reported in major studies—such as those by Biz et al.—on minimally invasive surgery (MIS), which have demonstrated that MIS Akin osteotomies are “safe, effective, and reliable” procedures, direct comparison between the results of the present study and those of studies investigating purely MIS techniques or hybrid MIS/open procedures may be methodologically inappropriate [34].

Direct comparison should be interpreted with caution, as the techniques used differ in several methodological and technical aspects beyond the surgical approach itself. Open and MIS procedures may differ in osteotomy technique and geometry, preservation of the lateral cortical hinge, fixation method, soft-tissue and periosteal disruption, and postoperative management. Therefore, differences observed between open and MIS series cannot necessarily be attributed to the surgical approach alone, and direct comparisons should ideally be performed in prospective studies with comparable patient populations, surgical indications, and outcome measures.

In this context, we believe that the present study represents an important step toward bridging the existing gap between the literature on conventional open surgery and that on MIS. Furthermore, it may provide a basis for future investigations, such as finite element analyses, which—by minimizing methodological bias—could enable more objective comparisons between different surgical techniques.

## 5. Conclusions

The absence of internal fixation does not adversely affect the maintenance of correction angles of the Akin osteotomy. The asymptomatic rate of delayed consolidation and asymptomatic rate of malunion were higher in the no-fixation group but this may be considered marginal and did not require correction treatment or variation in postoperative protocols. Preservation of the lateral cortex appears to be a critical factor for successful union in fixation-free Akin osteotomies. Surgeons should take special care to protect the lateral cortex during osteotomy. The results are promising; however, we believe that further studies are needed to confirm the data.

## Figures and Tables

**Table 1:** Descriptive statistics for patient characteristics are expressed as mean and standard deviation (SD) for continuous variables and absolute frequency and percentage (%) for categorical variables. The *p*-value is derived from the *t*-test for independent samples for continuous variables and the Chi-square test for categorical variables.

Patient Characteristics	FFA	SFA	*p*-Value
	*n* = 74	*n* = 68	
**Number of feet**	100	100	/
**Age, mean (SD)**	61.0 (11.5)	59.0 (12.0)	0.314
**Gender, *n* (%):**			0.169
**F**	67 (90.5%)	66 (97.1%)	
**M**	7 (9.46%)	2 (2.94%)	
**Diabetes, *n* (%):**			1.000
**No**	68 (91.9%)	63 (92.6%)	
**Yes**	6 (8.11%)	5 (7.35%)	
**Rheumatitis, *n* (%):**			1.000
**No**	71 (95.9%)	65 (95.6%)	
**Yes**	3 (4.05%)	3 (4.41%)	
**Smoking habits, *n* (%):**			0.426
**Not a smoker**	72 (97.3%)	64 (94.1%)	
**Smoker**	2 (2.70%)	4 (5.88%)	

**Table 2:** Descriptive statistics for IMA, HVA, DASA, IPA and IPOA expressed as mean and standard deviation (SD) for the FFA and SFA group at baseline (T0) and after surgery (T1) and the differences between them (Δ). Negative values for DASA and IPOA indicate correction toward varus (i.e., improvement).

	FFA (n = 100)		SFA (n = 100)		FFA (n = 100)	SFA (n = 100)
	T0	T1	T0	T1	Δ (T1 vs. T0)	
**IMA**	13.8 (3.15)	5.76 (2.70)	14.1 (2.80)	6.22 (2.38)	8.01 (3.01)	7.85 (2.58)
**HVA**	34.0 (9.55)	10.1 (7.02)	33.8 (10.0)	12.0 (6.64)	23.9 (7.98)	21.8 (8.27)
**DASA**	0.18 (4.17)	−6.22 (5.24)	3.88 (3.75)	−3.57 (4.21)	6.40 (5.02)	7.45 (5.22)
**IPA**	7.03 (8.87)	13.2 (4.97)	8.02 (5.05)	11.8 (6.20)	−6.18 (8.19)	−7.90 (6.48)
**IPOA**	7.42 (5.23)	−2.94 (6.34)	9.43 (5.51)	−0.33 (5.23)	10.40 (6.59)	8.35 (5.79)

**Table 3:** Mixed-effects models with fixed and random effects for procedures (id). Group (β1) represents the average difference in the response variable between the two groups at a given time. β2 represents the average change in the response variable for a unit change over time, holding the group constant. The interaction time * group represents how the change varies over time between the groups. SE = Standard Error. Significance code: * *p* < 0.01; ** *p* < 0.05; *** *p* < 0.001.

	IMA	HVA	DASA	IPA	IPOA
**Group (β1): SFA vs. FFA**	0.30	−0.21	3.70 ***	0.99	2.01 **
**SE**	(0.39)	(1.19)	(0.62)	(0.91)	(0.79)
**Time (β2): T1 vs. T0**	−8.01 ***	−23.90 ***	−6.40 ***	6.18 ***	−10.36 ***
**SE**	(0.39)	(1.19)	(0.62)	(0.91)	(0.79)
**Interaction (β3)**	0.16	2.11	−1.05	−2.42 *	0.60
**SE**	(0.55)	(1.69)	(0.88)	(1.29)	(1.12)

**Table 4:** Descriptive statistics for patient SFA and FFA complications expressed as absolute frequency and percentage (%). The *p*-value is derived from the Chi-square test (*n* ≤ 5) or the Fischer exact test (*n* ≤ 5).

	SFA	FFA	*p*-Value
	*n* = 100	*n* = 100	
Lateral Cortex Interrup:			<0.001
N	85 (85.0%)	58 (58.0%)	
S	15 (15.0%)	42 (42.0%)	
Delayed Union:			0.013
N	99 (99.0%)	90 (90.0%)	
S	1 (1.00%)	10 (10.0%)	
No Union:			1.000
N	99 (99.0%)	100 (100%)	
S	1 (1.00%)	0 (0.00%)	
Excessive Callus:			1.000
N	99 (99.0%)	100 (100%)	
S	1 (1.00%)	0 (0.00%)	
Malunion:			0.029
N	100 (100%)	94 (94.0%)	
S	0 (0.00%)	6 (6.00%)	

**Table 5:** Association between lateral cortex interruption and post-surgical complications (delayed union and malunion) in SFA and FFA groups. Values are presented as absolute frequencies and percentages. The *p*-values are derived from the Chi-squared test.

		SFA			FFA		
		Lateral Cortex Interrup			Lateral Cortex Interrup		
		N	S	*p*-Value	N	S	*p*-Value
**Delayed Union**	N	84 (98.8%)	15 (100%)	0.035	56 (96.6%)	34 (81.0%)	0.026
	S	1 (1.18%)	0 (0.00%)		2 (3.45%)	8 (19.0%)	
**Malunion**	N	85 (100%)	15 (100%)	/	57 (98.3%)	37 (88.1%)	0.091
	S	0 (0.00%)	0 (0.00%)		1 (1.72%)	5 (11.9%)	

## Data Availability

The data presented in this study are available on request from the corresponding author due to privacy.
